# Coincidence Detection of Place and Temporal Context in a Network Model of Spiking Hippocampal Neurons

**DOI:** 10.1371/journal.pcbi.0030234

**Published:** 2007-12-14

**Authors:** Yael Katz, William L Kath, Nelson Spruston, Michael E Hasselmo

**Affiliations:** 1 Interdepartmental Biological Sciences Program, Northwestern University, Evanston, Illinois, United States of America; 2 Department of Applied Mathematics, Northwestern University, Evanston, Illinois, United States of America; 3 Department of Neurobiology and Physiology, Northwestern University, Evanston, Illinois, United States of America; 4 Center for Memory and Brain, Program in Neuroscience, Boston University, Boston, Massachusetts, United States of America; University College London, United Kingdom

## Abstract

Recent advances in single-neuron biophysics have enhanced our understanding of information processing on the cellular level, but how the detailed properties of individual neurons give rise to large-scale behavior remains unclear. Here, we present a model of the hippocampal network based on observed biophysical properties of hippocampal and entorhinal cortical neurons. We assembled our model to simulate spatial alternation, a task that requires memory of the previous path through the environment for correct selection of the current path to a reward site. The convergence of inputs from entorhinal cortex and hippocampal region CA3 onto CA1 pyramidal cells make them potentially important for integrating information about place and temporal context on the network level. Our model shows how place and temporal context information might be combined in CA1 pyramidal neurons to give rise to splitter cells, which fire selectively based on a combination of place and temporal context. The model leads to a number of experimentally testable predictions that may lead to a better understanding of the biophysical basis of information processing in the hippocampus.

## Author Summary

Understanding how behavior is connected to cellular and network processes is one of the most important challenges in neuroscience, and computational modeling allows one to directly formulate hypotheses regarding the interactions between these scales. We present a model of the hippocampal network, an area of the brain important for spatial navigation and episodic memory, memory of “what, when, and where.” We show how the model, which consists of neurons and connections based on biophysical properties known from experiments, can guide a virtual rat through the spatial alternation task by storing a memory of the previous path through an environment. Our model shows how neurons that fire selectively based on both the current location and past trajectory of the animal (dubbed “splitter cells”) might emerge from a newly discovered biophysical interaction in these cells. Our model is not intended to be comprehensive, but rather to contain just enough detail to achieve performance of the behavioral task. Goals of this approach are to present a scenario by which the gap between biophysics and behavior can be bridged and to provide a framework for the formulation of experimentally testable hypotheses.

## Introduction

The hippocampal network needs to integrate information about place and temporal context to enable an animal to navigate its environment based on previous experience [1–5]. Since the discovery of place cells, which fire selectively when a rat is in a particular location [6], it has been clear that the hippocampus encodes information about space. More recently, experiments have pointed to additional components of spatial representation in the rat hippocampus. In a spatial alternation task on a T-maze, some CA1 cells fire when the rat is in a particular location on the stem of the maze, but only after either a left-turn or a right-turn trial [1]. A majority of cells respond on the basis of recent history, though some are predictive of future action [7]. These cells, sometimes referred to as “splitter cells” or “episodic cells” [1,7–9], are thought to be neural correlates of temporal context. The term “context” can be operationally defined in many other ways [2], including more temporally diffuse effects defining an extended period of behavior or a specific goal [10], or nontemporal effects such as overall environment or presence of specific cue stimuli [11]. In this paper, we consistently use the phrase “temporal context” [12] to refer specifically to the history corresponding to one lap on the alternating T-maze.

A previous model [2] analyzed how splitter cells might emerge in the hippocampus during spatial alternation using the effect of temporal context [12] and based on other behavioral and physiological data available on the hippocampal formation. That model reproduced the splitter-cell phenomenon, but the result depended upon a multiplicative interaction between the two major inputs to CA1 pyramidal neurons: the perforant-path input from layer III of entorhinal cortex (ECIII) and the Schaffer-collateral input from CA3. At the time the model was made, the idea that a nonlinear interaction between these two inputs was required to produce CA1 output was an assumption, lacking a biophysical basis.

Recently, it was discovered that inputs from layer III pyramidal cells of entorhinal cortex, which selectively target the distal dendrites of CA1 pyramidal cells, interact nonlinearly with inputs from CA3 pyramidal neurons (CA3), arriving more proximally [13]. Distal inputs alone typically generate dendritic spikes, but these spikes fail to propagate to the action potential initiation zone in the axon. If a subthreshold depolarization of the proximal dendrites arrives in the same time window as distal dendritic spikes, however, the more proximal input can facilitate propagation of the dendritic spike, resulting in generation of an axonal action potential. This biophysical interaction can be regarded as “gating” of the dendritic spike by the CA3 input. This suggests that CA1 pyramidal cells can act as coincidence detectors.

The previous model [2] could not immediately be employed to examine whether gating in CA1 pyramidal neurons might provide the necessary multiplicative interaction at the network level because it uses firing rates as opposed to individual spiking units. In this study we therefore constructed such a spiking model, using reduced models of CA1 pyramidal neurons that exhibit gating, and we show how this model can produce activity for guiding the trajectory of a rat in the simulated spatial alternation task.

Our model incorporates several biophysical considerations into a successful algorithm for simulating the spatial alternation task. Gating in CA1 dendrites gives rise to splitter cells, and the output of CA1 neurons is used to guide the rat's trajectory through the maze. Thus, we show directly how concerted behavior could emerge from the detailed cellular properties of hippocampal and entorhinal neurons. Our model also points to requirements for a neural representation of temporal context and suggests how the sources of place and temporal context representations could be identified experimentally.

## Results

Three regions of the hippocampus were simulated: ECIII, CA3, and CA1. The network consists of representations of cells in each region and their excitatory synaptic interconnections.

### ECIII and CA3 Neurons

ECIII and CA3 neurons were modeled as single nodes (equipotential compartments) using the equations proposed by Izhikevich for quadratic integrate and fire neurons with adaptive recovery and voltage reset [14]. Single nodes were sufficient to represent ECIII and CA3 pyramidal neurons because we were not concerned with dendritic processing in those cells. The Izhikevich scheme was chosen because it is simple, computationally efficient, and capable of reproducing a wide range of neuronal behaviors.

### CA1 Neurons

**Figure 1:**
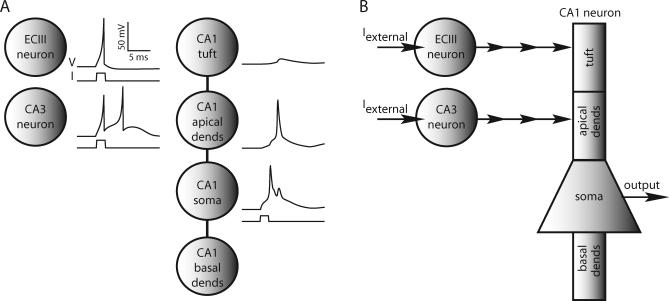
Elements of the Network Model (A) ECIII and CA3 neurons are represented by single ECIII and CA3 nodes. Reduced model of a CA1 pyramidal neuron consists of four CA1 nodes electrically coupled together, representing the apical tuft, more-proximal apical dendrites (dends), soma, and basal dendrites. Shown are voltage responses of single, uncoupled ECIII, CA3, and CA1 neurons to 2-ms current injections of 200 pA, 200 pA, and 375 pA, respectively. The backpropagating action potential into the apical dendritic compartments of our CA1 pyramidal cell model shows that it has weakly excitable dendrites. (B) ECIII and CA3 cells receive external current inputs during the simulations. An ECIII cell provides input to the distal dendritic compartment of a CA1 cell, and a CA3 cell innervates its proximal dendritic compartment. Synaptic potentials are modeled as alpha functions, and if the voltage in the presynaptic cell exceeds −30 mV, an input is given to the postsynaptic cell.

Multiple nodes were required to represent CA1 neurons in order to simulate gating, which is a result of the geometry and excitability of their dendritic trees. We used a conductance-based model for the CA1 cells to make connection with our previous multicompartmental models that exhibited gating [13]. CA1 neurons were each composed of four CA1 nodes, corresponding to the distal apical tuft, apical dendrites, soma, and basal dendrites of a CA1 pyramidal cell. These nodes were electrically coupled together in a manner corresponding to pyramidal neuron geometry (Figure 1). The areas of the nodes were approximately scaled to the areas of the regions they represent in the multicompartmental model of a reconstructed CA1 pyramidal neuron [15]. In the multicompartmental model, channel densities were adjusted to match experimental data, so in our reduced model, we use similar densities (see Methods for model equations). The response of our reduced model CA1 neuron to a somatic current injection (Figure 1A) illustrates that it has weakly excitable dendrites with the backpropagating action potential failing to invade the distal dendrites, as in the full morphological models and in experiments [15].

### The Virtual Environment

**Figure 2:**
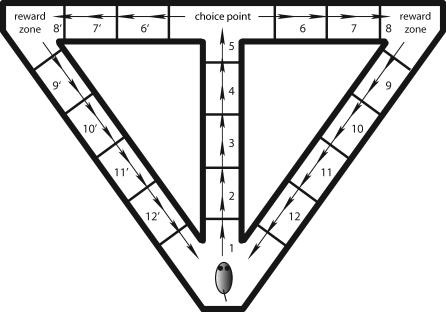
The Virtual Environment The virtual rat is confined to move through a T-maze with return arms. Although it moves in small steps, the maze is divided into larger positions, numbered 1–5 for positions on the stem, 6–12 for positions on the right, and 6′–12′ for corresponding positions on the left. The rat begins in position 1 at the base of the stem, moving up the stem to the choice point at the top of the stem. Virtual reward zones are in the right and left corners of the maze. The arrows denote a correct trajectory with the rat alternating between right and left turns at the choice point.

The virtual rat is confined to move through a T-maze with return arms (Figure 2). It begins at the base of the stem, and at every time step, updates its position by an amount equal to Δ*x*. Although the rat moves with small steps, the maze is also divided into larger positions, marked in the figure. The first time through the maze, the rat is forced to take an alternating trajectory marked by the arrows. On all subsequent runs, the rat chooses where to go by following the spiking patterns of its CA1 neurons, as discussed below.

The objective of the spatial alternation task is for the rat to earn rewards, which the experimenter alternately places in the top right and left corners of the maze. In the model, the rewards are not explicitly simulated, but a trial is considered correct if the rat runs to the reward zone that would have contained the reward in the actual task (Figure 2). On each trial, the rat runs from the base of the stem to the position marked “choice point” where it must decide which way to turn. A correct choice requires the rat to remember which way it turned on the previous trial, so it can head toward the opposite reward zone.

Many areas of the hippocampal formation are known to contain place cells, but where the place representation originates in the brain is not fully understood. Similarly, although the hippocampus is known to represent temporal context, the origin of this representation has not been identified. Therefore, we test two model variants: In the first, we assume that primary place information is represented in ECIII and temporal context is represented in CA3. In the second, we assume the reverse, that primary place information is represented in CA3 and temporal context in ECIII.

### Forward Association

Each position in the environment is represented by one primary place cell, which receives an external current input every time the rat enters a particular position. The primary place cells are either ECIII cells or CA3 cells, depending on which region is assumed to contain the raw representation of place in the particular simulation.

**Figure 3:**
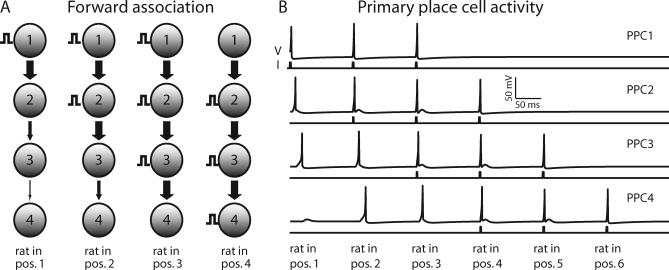
The Network of Primary Place Cells (A) First column: when the rat enters position 1, PPC1 receives an external input. *w* factors are decreased (see text) so that the input propagates forward to PPC3, but the response in PPC4 is below spike threshold. Second column: when the rat enters position 2, all *w* factors are reset. PPCs 1 and 2 receive external inputs that elicit spiking in PPCs 3 and 4, but not PPC5. When the rat enters positions 3 and 4, external inputs are delivered and *w* factors are adjusted in a similar manner (remaining columns). (B) Time series plots for the primary place cells representing positions 1, 2, 3, and 4 on the maze. For each cell, the bottom trace is the input current and the top trace is the voltage response. PPC*x* gets external input at positions *x*, *x* + 1, and *x* + 2, and forward association input from positions *x* − 1 and *x −* 2. Therefore, a PPC spikes at most in five positions.

We assume that at the start of the simulation, the rat has already learned the spatial alternation task, so the appropriate network connectivity has been established. Every primary place cell is synaptically connected only to those primary place cells representing the positions that the rat can enter from its current position. Thus, cell 1 is connected to cell 2, cell 2 to cell 3, and so forth (Figure 3A); this is termed forward association. When the rat is at the choice point, it can turn either right or left; cell 5, therefore, is connected both to cells 6 and 6′.

In the real brain, excitatory inputs do not typically propagate through entire networks because of the requirement for inputs from many cells to drive spiking and the abundance of inhibitory inputs [16–18]. In our model, we limit the spread of activity through the network of primary place cells by decreasing the factor *w* in the transfer function between cells (see Methods) by 60% for each successive connection. For the first connection, *w* is at a maximum value (*w_max_*), which is sufficient to always induce spiking in cells directly connected to the primary place cell receiving input. To prevent inputs from exciting the entire network, we decrease *w* with distance from the input site. Reducing *w* for every connection does not allow for sufficient membrane depolarization to bring the third cell in the chain to firing threshold. For example, if cell 1 receives an input, the connection from cell 1 to cell 2 has a weight of *w_max_*, the connection from cell 2 to cell 3 has a weight of 60% of *w_max_*, which is sufficient to cause cell 3 to fire, and the connection from cell 3 to cell 4 has a weight of 60% of 60% of *w_max_*, which is not sufficient to bring cell 4 to threshold. Every time the rat enters a new position, the *w* factors are adjusted so that the forward connections follow this pattern (Figure 3A). This mechanism is not intended to directly model any biological process. Rather, it is a simple phenomenological way of limiting the forward spread of activity through the network without explicitly including more complex effects such as inhibition and stochastic firing of neurons.

When the rat enters a new position, the primary place cell (PPC) representing that position receives an external current input representing place information. The PPC representing that position continues to get external input when the rat moves to the next two locations. Combining this system with forward association results in place fields that span five positions, which are larger than the spatial elements in our model [19]. The size of the model place fields is reasonably consistent with the size of experimentally observed place fields [20]. This scheme also mimics the fact that in vivo, place cells fire on several theta cycles once they are activated [21].

**Figure 4:**
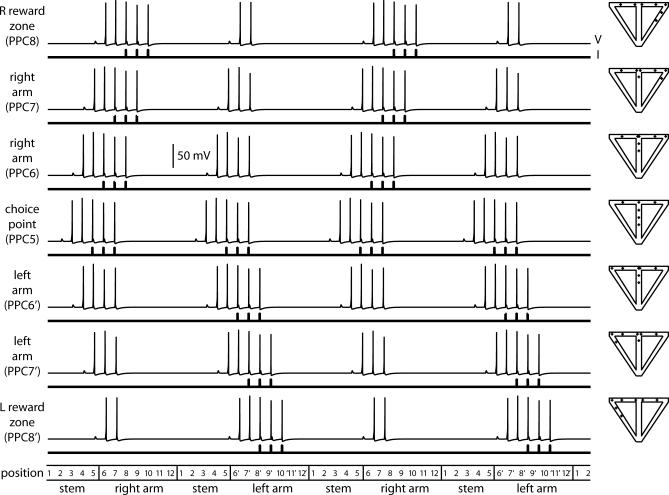
Forward Association from the Choice Point Left: time-series plots for the PPCs representing the choice point and the three positions to the right and left of the choice point. For each cell, the bottom trace is the input current (200 pA) and the top trace is the voltage response. Activity spreads symmetrically from the choice point cell to the cells representing the right and left arms of the maze. Right: place fields for the cells depicted on the left. The dots represent spikes, showing where the animal was located when the cell fired.

When the rat is in the start position at the base of the stem, primary place cell 1 (PPC1) receives an external input. PPC1 then fires, and forward association results in firing of PPC2 and PPC3 and an excitatory postsynaptic potential (EPSP) in PPC4 (Figure 3B). When the rat moves up the stem into position 2, PPCs 1 and 2 receive external input, and the spike in cell 2 propagates through PPC4. When the rat gets to the choice point at the top of the stem, PPC5 gets external input that spreads both to the right to PPCs 6 and 7 and to the left to PPCs 6′ and 7′ (Figure 4). If the rat turns to the right and enters position 6, PPCs 6′ and 7′ on the left will remain firing because PPC5 at the choice point continues to receive input. Once the rat reaches position 8, the right reward zone, the forward association from the choice point to PPCs 6′ and 7′ stops, and only cells in front of the rat fire. Since the firing of the choice point cell spreads symmetrically to both the right and the left arms of the maze, the rat must use temporal context information to choose the correct trajectory.

### Temporal Context

**Figure 5:**
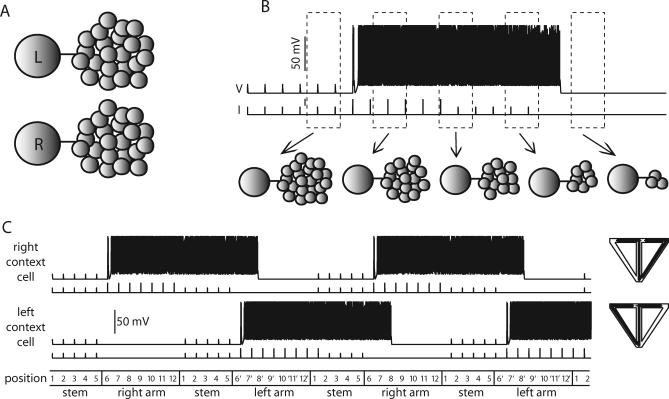
The Network of Temporal Context Cells (A) At first, the left (L) and right (R) TCCs are each connected to large recurrent networks of 22 cells each. (B) When the rat enters the right arm of the maze, the right TCC receives strong external input (200 pA), causing it to fire. With successive spiking, the right TCC can recruit a smaller and smaller portion of its network. The TCC continues to fire without external input only as long as it can recruit a recurrent network of sufficient strength. (C) Left: time-series plots for the right and left TCCs. Bottom traces are the input current, and top traces are the voltage response. Note that the magnitude of the input current increases (from 100 to 200 pA) as the rat moves from the stem into the arms of the maze. Right: context–place fields for the cells on the left. Note that the context–place fields are extremely broad.

Our model utilizes two temporal context cells with very broad place fields to encode temporal context; one represents the stem and the left half of the environment, and the other represents the stem and the right half (Figure 5A). In our model, a temporal context cell (TCC) is a place cell whose firing outlasts the external input, but is not sustained forever. Such sustained neuronal firing is the fundamental requirement for a representation of temporal context [12]. There are several mechanisms available both on the single-cell and network levels that could give rise to it [12], and in our model, we choose a recurrent network for simplicity. The first time a TCC fires, it activates a large network that feeds back onto itself, and as it fires successive spikes, the percentage of the network that it succeeds in recruiting decreases (Figure 5B). Specifically, the recurrent network for each TCC contains 22 neurons, and for every 40 spikes fired, the number of network cells activated is decreased by one. This has the effect of keeping a TCC firing for a limited amount of time after input to it has ceased.

As the rat enters each position on the stem of the maze, both TCCs receive an external input that is too weak to induce firing in either cell (Figure 5C). If it makes a right turn, the input to the right TCC increases, causing it to fire, but the input to the left TCC ceases. When the rat reenters the stem after the right turn, both TCCs receive weak input again, but this is sufficient to keep the right TCC firing, but not to initiate firing of the left TCC. Furthermore, the right TCC continues to fire for several positions after the rat has made a left turn even though input to it has ceased. Thus, when the rat turns left after a preceding right turn run, the right TCC is still spiking and the left temporal context cell has not yet begun to fire. As the rat continues to move through the left arm of the maze, the right TCC shuts off and the left one begins to fire. This lateral selectivity of the right and the left TCCs is used by the virtual rat to determine which way to turn.

### Computation by CA1 Neurons

Each position in the maze is also represented by two CA1 neurons. The model CA1 neurons have just four compartments, but are capable of reproducing the gating phenomenon [13]. In our model, input from ECIII enters the distal dendritic compartments of the CA1 cells, mimicking the perforant-path input that selectively innervates the apical tufts of CA1 pyramidal neurons, and input from CA3 enters their more proximal dendritic compartments, mimicking the Schaffer-collateral input. On their own, the ECIII inputs generate dendritic spikes in the CA1 tuft, which fail to propagate forward to the soma. The CA3 inputs on their own generate EPSPs in the proximal apical dendritic compartment of the CA1 neurons, but are insufficient to induce spiking. When the ECIII and CA3 inputs are coincident, however, propagation of the dendritic spike is rescued, resulting in somatic action potentials.

**Figure 6:**
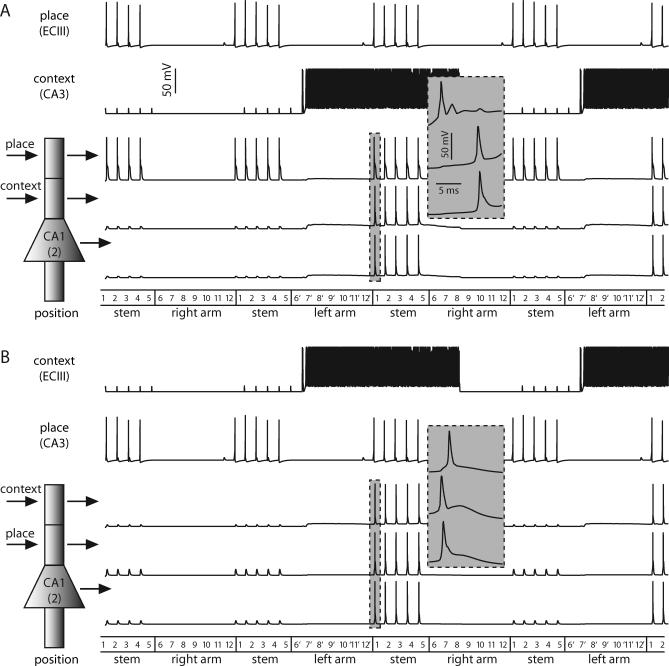
Gating in the Reduced Model of a CA1 Pyramidal Neuron (A) Shown are the cells representing position 2 of the maze. Here, the ECIII cell encodes place information and the CA3 cell represents temporal context. The CA1 cell only fires somatic spikes when the ECIII and CA3 inputs are coincident. As the rat enters the stem from the right arm, the subthreshold responses in the proximal apical dendrites and soma correspond to dendritic spikes that fail as they propagate forward. The gray inset shows the first set of CA1 spikes on an expanded time scale. (B) Same as above except the ECIII cell represents temporal context and the CA3 cell encodes raw place information. Although the somatic action potential profiles in (A) and (B) are roughly identical; in this case, the spike is initiated in the proximal apical dendritic compartment and propagates forward to the soma and backward to the apical tuft, as can be seen in the gray inset.

If we assume the ECIII cells are PPCs and the CA3 cells are TTCs, the CA1 neurons fire dendritic spikes in their most distal nodes and experience sustained depolarization of their more proximal ones, but fire somatic spikes only when both the place cells and the TCCs are coactive. This case corresponds to gating, because the spike is initiated in the apical tuft and propagates forward to the soma because of the extra depolarization entering the more proximal region (Figure 6A).

If we assume that the PPCs occur in CA3 and TCCs in ECIII, the output of the CA1 neuron is the same as in the previous case because we require both the ECIII and CA3 inputs for spiking (Figure 6B). In our reduced model, the persistent input to the CA1 apical tuft compartment due to the ECIII temporal context cell serves to depolarize the apical tuft for long periods of time. This depolarization sums with the depolarization entering more proximally, bringing the apical dendritic compartment past action potential threshold. With a different choice of parameters in our model, the action potential could have been initiated in the soma instead of the proximal apical dendrites, but in either case, the action potential readily spreads throughout the rest of the cell. The facilitated spike propagation in the dendrites (compare Figures 1 and 6) results from the synaptic depolarization associated with activation of the Schaffer-collateral input.

### CA1 Output Guides the Trajectory of the Rat

In our model, the rat uses the output of its hippocampus to select actions at all locations in the maze. Action selection in spatial memory tasks is a complex process involving interactions of the hippocampus with the prefrontal cortex and other regions, which receive hippocampal output as their input. Instead of trying to simulate these dynamics, we use a simple rule by which action selection is determined from the output of the hippocampus directly: the rat always moves to a position corresponding to a spiking CA1 neuron, with the stipulations that it can only move to an adjacent position and it cannot move backward.

**Figure 7:**
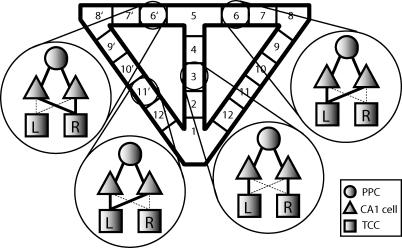
Network Wiring Diagram Circles represent PPCs, triangles represent CA1 cells, squares represent TTCs cells, and lines indicate connections. The solid lines show robust connections that came about as a result of a presumed learning process, whereas the dashed lines suggest weak connections that have not been strengthened due to learning. Cells representing positions in the stem of the maze are connected as depicted for position 3: one CA1 cell for position 3 is connected to PPC3 and the right (R) TCC, whereas the other CA1 cell representing position 3 is connected to PPC3 and the left (L) TCC. Cells representing positions in the arms of the maze (except for positions on either side of the choice point, see below) are connected in the same way as the cells for position 11′. Both CA1 cells representing position 11′ are connected to PPC11′ and to the TCC representing the ipsilateral side of the maze, in this case the left TCC. The exception to this occurs in positions 6, 7, 6′, and 7′, which are wired as follows: both CA1 cells representing each position are connected to PPC representing that position and to the TCC representing the opposite side of the maze from which the position is located.

**Figure 8:**
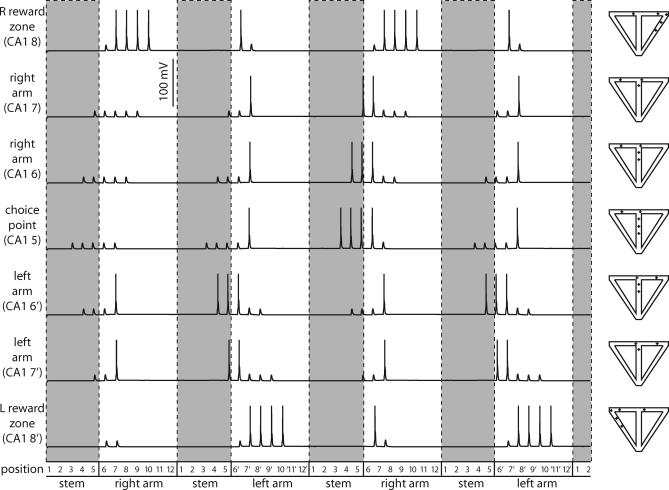
Firing Patterns of CA1 Cells near the Choice Point Left: time-series data for the somata of CA1 neurons representing the choice point and the three positions to the right and left of the choice point. There are two CA1 cells associated with each location, as described in the text; only one cell for each location is shown. For the cell representing the choice point (CA1 5), the cell shown is the one associated with a right turn. Here, PPCs are taken to be ECIII cells, and TCCs to be CA3 cells, but when the representations are switched, the time series is essentially unchanged. Note that this figure includes the first time the rat goes through the maze, so it contains the initial transient where the final dynamics of all the neurons have not yet been established. Right: place fields for the somata of the cells on the left.

For this single rule to govern the movement of the rat through the entire task, the wiring of the network was set up as follows. The two CA1 cells representing each position in the maze receive input from the PPC representing that position and from both TCCs (Figure 7). Although both TCCs project to every CA1 cell, we presume that some learning process has taken place to strengthen some connections and weaken others. Thus, for positions on the stem of the maze, one CA1 cell receives strong input from the right TCC and weak input from the left one, and the other receives strong input from the left TCC and weak input from the right one. CA1 cells for the right return arm of the maze (positions 8–12) receive strong input from the right TCC, and CA1 cells for the left return arm of the maze (positions 8′–12′) receive strong input from the left TCC. For the two positions adjacent to the choice point on either side, the situation is reversed: CA1 cells 6 and 7 on the right side of the maze receive strong input from the left TCC, and CA1 cells 6′ and 7′ on the left side of the maze receive strong input from the right TCC (Figure 7). This enables the rat to move simply by following the spiking of its CA1 neurons. For example, if the rat is at the choice point and it has previously completed a right-turn run, CA1 cell 6′ will be spiking, but cell 6 will not. Based on this information, the rat will enter position 6′ and move toward the reward zone on the left side of the maze (Figure 8). Thus, with biophysically realistic elements wired together in this manner, a simple rule is sufficient to simulate the spatial alternation task.

### Simulation of Splitter Cells

**Figure 9:**
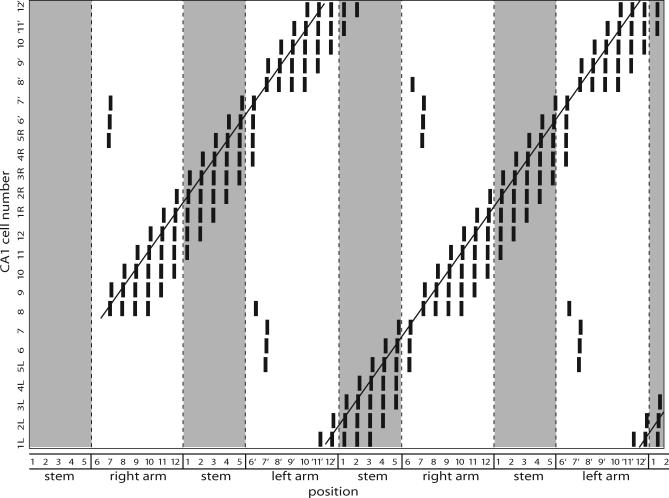
The CA1 Network Raster plot showing spiking patterns for the entire CA1 network (including the initial transient). Cell number is plotted against position, and a vertical bar indicates a somatic spike when the rat is in a particular position. Cells in the stem are splitter cells: CA1 cells 1–5 R and 1–5 L fire only after right-turn and left-turn trials, respectively. The lines show how the rat can use the output of its CA1 cells to determine correct trajectories through the maze.

The interaction of place and temporal context inputs to cells representing locations in the stem effectively results in splitter-cell responses (Video S1). Figure 9 illustrates the output of CA1 neurons representing all positions in the maze. When the virtual rat enters the stem from the right arm, the network shows clear firing activity in one set of neurons representing the stem (1R, 2R, 3R, 4R, and 5R), but not in the other set of neurons representing the stem (lack of activity in 1L, 2L, 3L, 4L, and 5L). In contrast, when the virtual rat enters the stem from the left arm, the network shows firing activity in a different set of neurons representing the stem (1L, 2L, 3L, 4L, and 5L), and does not show firing activity in the previously active set of neurons representing the stem. This demonstrates that the cellular gating phenomenon used by the model CA1 cells provides the necessary mechanism for selective firing based on prior temporal context.

In summary, we have shown how a differential representation of temporal context in the hippocampus might be constructed from the biophysics of hippocampal and entorhinal pyramidal neurons. The CA1 cells in the stem of the maze are place cells, but they also fire selectively based on temporal context. One population of CA1 cells in the stem fires only after left-turn trials, and the other fires only after right-turn trials (Figure 9). This is a direct consequence of a nonlinear interaction between the ECIII and CA3 inputs, causing the CA1 cells only to fire if they get coincident input from these two pathways. Because one population of CA1 cells in the stem is strongly connected to the right TCC and the other to the left TCC, the CA1 cells in the stem become splitter cells.

## Discussion

### Representations of Context in the Rat Hippocampus

Although studies in humans suggest that the role of the hippocampus in episodic memory requires context for where and when an event occurs [22], the idea that the representation of space in the rat hippocampus includes a contextual component remains somewhat controversial. Early evidence for a hippocampal representation of context comes from the observation that some place cells are active only when a rat is traveling in a particular direction in tasks such as the radial maze or linear track, but not when the rat is running on an open field [20,23]. Place cells also remap their firing locations when a rat searches for food in a directed manner as opposed to foraging randomly [9]. These data indicate that not only does the hippocampus encode locations, but the representation changes depending on the behavioral context.

Additional evidence for a contextual component of spatial representation in the hippocampus comes from the discovery of splitter cells, CA1 place cells that fire only after a left- or a right-turn trial in a spatial alternation task [1,7,8]. Splitter cells were not observed in spatial alternation on a Y-maze [24]; later experiments, however, showed that a reward presented at the base of the stem prevents the splitter-cell phenomenon, and splitter cells are observed if a reward is not presented at the start of the overlapping segment [25].

Behavioral data show that hippocampal lesions impair a rat's performance of spatial alternation when a delay is imposed between right-turn and left-turn trials, but do not impair its performance of the task when it alternates through the maze continuously [26,27]. Recent recording experiments show that context-dependent hippocampal activity occurs in both the delayed and continuous versions of the spatial alternation task, although, paradoxically, in the delayed version, it occurs during the delay period and not on the stem of the maze [27]. Thus, although the hippocampus is not required for continuous spatial alternation, it generates splitter-cell activity during the task. The differences in hippocampal activity during the delayed and continuous versions of spatial alternation indicate that the hippocampus is a dynamic system that may adapt to the demands of different tasks [27].

Another study shows that neurons recorded in the same spatial location, but in recording chambers with different shapes, have firing rates differing by several orders of magnitude, whereas their place fields remain the same. Conversely, neurons recorded in recording chambers of the same shape, but in different spatial locations, show a change in both the rate and location of firing [11], indicating that the hippocampus contains codes for both spatial position relative to local cues and the context of the overall location of the local cues in the environment.

Although it now seems clear that the hippocampus represents context, the origin of the contextual representation in the hippocampal network is not known. In our model, a requirement for a representation of temporal context is a transient response that outlasts the stimulus that generated it (e.g., a right turn) but is not sustained forever. In different versions of our model, we incorporated this in ECIII or CA3 neurons, under the assumption that each cell type has the potential to perform that function. ECIII neurons have been shown to exhibit sustained firing that could be manipulated by varying their inputs [28,29]. The representation of temporal context by a gradual reduction in sustained neural activity used here was based on previous models of temporal context [12,30,31]. A distinguishing anatomical feature of the CA3 network is that CA3 pyramidal cells are reciprocally connected to one another [32], which could enable them to continue spiking long after input to them has ceased [33]. Since either single neurons in ECIII [28,29] or the recurrent network connectivity in CA3 could instantiate the representation of temporal context in the real hippocampus, we represented temporal context in these two ways in different versions of our model. Although both models were able to reproduce splitter cells in CA1, the responsible biophysical interaction was slightly different in the two models.

### Predictions of the Model

Our model predicts different behavior in CA1 cells depending on which of its afferents carry temporal context information. If temporal context enters CA1 from CA3, its function is to facilitate forward propagation of dendritic spikes triggered by the place information arriving in the distal tuft via the ECIII input (Figure 6A). If temporal context enters CA1 from entorhinal cortex, it depolarizes the apical dendrites and facilitates a spike in response to the place information arriving in more-proximal dendrites via the CA3 input. In this case, the action potential is initiated in the proximal region of the cell and backpropagates into the distal dendrites (Figure 6B). This is because in our model, the high-frequency input arriving from the TCCs causes a depolarization of the CA1 dendrite rather than causing a distal dendritic spike.

The model also makes a specific prediction that splitter-cell activity in CA1 requires inputs from both ECIII and CA3. Although inputs from CA3 to CA1 have been reduced or eliminated in a few studies [34–36], the effects of these manipulations on splitter cells have not been determined. However, the finding that CA1 place cells are not disrupted by elimination of CA3 inputs [36] is seemingly at odds with our model, which requires both CA3 and ECIII inputs to produce firing. This result could be explained, however, by an upregulation of ECIII innervation following CA3 lesions. Rapid and reversible inactivation of ECIII or CA3 inputs would provide more stringent tests of our model.

### Relation to Previous Models

There are many models of the hippocampus that attribute specific functions to individual subregions, and a few full models that attempt to integrate the functions of the different subregions [37,38]. The model presented here is related to a previous model of neural activity during spatial alternation [2], which effectively simulates the phenomenon of splitter cells due to a multiplicative interaction of ECIII and CA3 inputs to CA1 neurons. However, our model is fundamentally different from the previous one because in that model, activity was represented in a more abstract manner, using mean firing rates in hippocampal regions, rather than spikes in biophysically realistic neurons. In this study, we recast many aspects of the previous model into a spiking model constrained by experimental data. Another difference is that the previous model used single neurons to represent locations on the stem and obtained splitter-cell responses during retrieval through the differential activation of neurons representing the left or right reward arm. In contrast to the current model, the previous model showed more splitting, primarily near the choice point, and the presence of splitters at earlier points on the stem required the specification of very large place fields. The previous model also differed in that it modeled a learning-based development of the representation of space and temporal context, it incorporated theta rhythms, and it included an abstract representation of prefrontal cortex to guide behavior.

The model presented here addresses specific biophysical mechanisms important for solving problems that require the use of context. Earlier models have addressed different mechanisms for context-dependent changes in neural firing activity using more-abstract threshold units [39,40]. In other models, spiking network models of the hippocampus were developed to guide navigation toward different goal locations [41,42]. Our model complements these previous approaches by using more biophysically realistic models of neurons and relating these properties to the context-dependent properties of splitter cells.

### Limitations of the Model and Opportunities for Developing Anatomically and Biophysically Realistic Models of the Hippocampus

Our model is a very simple representation of place and temporal context in the hippocampus, intended primarily to highlight possible biophysical mechanisms by which these properties could be represented in ECIII and CA3, and mechanisms by which coincidence of these signals could lead to spiking in CA1 pyramidal neurons. Although simple models can offer insight and predictions, identifying some of the simplifying assumptions highlights the possibility of future enhancements to the model.

One simplification in the present model is the fact that we simulate only three of the many hippocampal regions likely to be important for delayed spatial alternation. Input to CA3 comes from ECII both directly and indirectly via the dentate gyrus, and information processing in these regions should be considered in future models.

Increasing the number of neurons could also enhance our model by allowing for a more continuous representation of space and a more distributed representation of temporal context. In addition, representing each location by a population of neurons would allow each cell to respond to its inputs stochastically, which would be a closer reflection of reality than our simple implementation.

Also not considered in our model are the prominent theta and gamma oscillations in the hippocampus believed to be important for spatial processing [21,23,43]. Oscillations are likely to be important for the encoding of place and context information, as well as for the synaptic plasticity that may underlie the dynamic nature of their hippocampal representation.

CA1 pyramidal neuron dendrites are innervated by several types of interneurons, which are not included in our model. As inhibition is likely to profoundly influence the integration of excitatory inputs from ECIII and CA3 as well as hippocampal oscillations, biophysically realistic models of hippocampal networks should certainly include such interneurons.

In our model, we assumed that learning has already taken place to establish the network wiring. Other models have addressed the process of encoding associations between sequentially active place cells [42,44,45]. Incorporation of these mechanisms could be used to study the mechanisms by which the connectivity we used in our model (e.g., forward association and cross-wiring) could be established.

Another simplification of our model is that primary place and temporal context information are represented separately in ECIII or CA3. In reality, however, there is evidence for representations of space in both CA3 [11,33,46,47] and EC [8,48,49]. In addition, transverse lesions to the dorsal CA3 region of rat hippocampus revealed impairments in spatial-memory retention in the Morris water-maze task [50], and selective CA3 lesions impair detection of novel spatial arrangements of objects [51]. Both of these studies suggest that CA3 can also encode different types of context during specific behavioral tasks. A more sophisticated model would therefore utilize hybrid place–context neurons in CA3 and possibly in ECIII as well.

These limitations represent opportunities for improvements and enhancements of our model. In addition, they highlight the need for the merger of cellular and systems-level studies of the hippocampus before a complete picture will emerge regarding the dynamic and complex representation of information in the hippocampus.

## Methods

The ECIII and CA3 node types use the equations due to Izhikevich for a quadratic integrate and fire neuron with adaptive recovery, and the rule that after a spike, the voltage, *v*, is reset to the parameter *c*, and the recovery variable, *u*, is incremented by the parameter *d* [14].

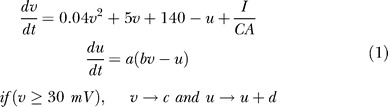



The model requires two other parameters: *a*, which represents the inverse time scale of *u*, and *b*, which represents the sensitivity of *u* to subthreshold changes in *v*. In all simulations, the parameters take the values *a* = 0.02 ms^−1^, *b* = 0.2, *c* = −65 mV, and *d* = 4 mV, which result in regular spiking behavior. When a node is designated as a TCC, however, the parameters are *a* = 1 ms^−1^, *b* = 0.2, *c* = −60 mV, and *d* = −20 mV, which produce a more prominent after-depolarization and increased excitability. All ECIII and CA3 cells are assumed to have an area of 1,000 μm^2^ and a capacitance of 1 μF/cm^2^.

The CA1 node types use Hodgkin-Huxley–style equations for sodium channels, delayed rectifier potassium channels, and A-type potassium channels.

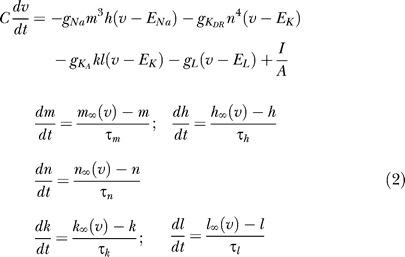



The model parameters are as in [15] and [52], and are listed in Tables 1 and 2.

**Table 1:**
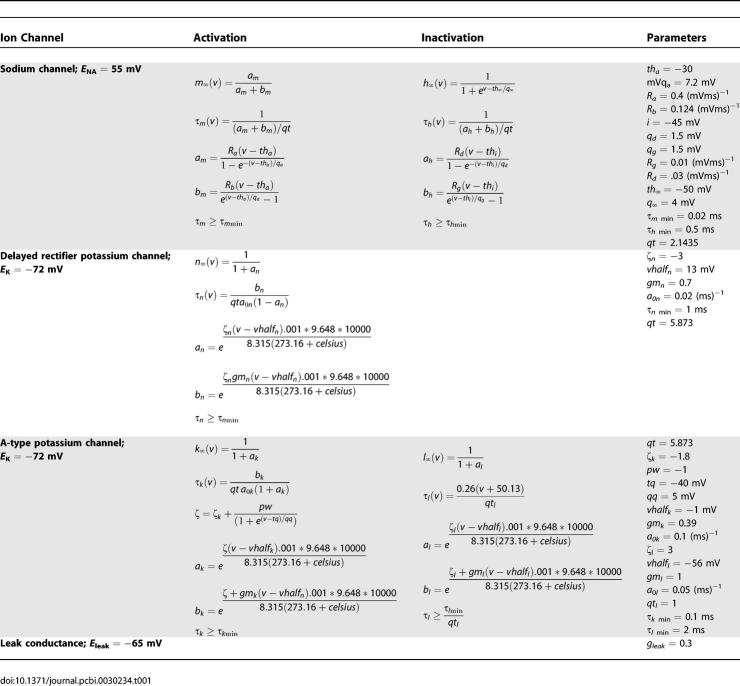
Parameters of CA1 Pyramidal Cell Models

**Table 2:**
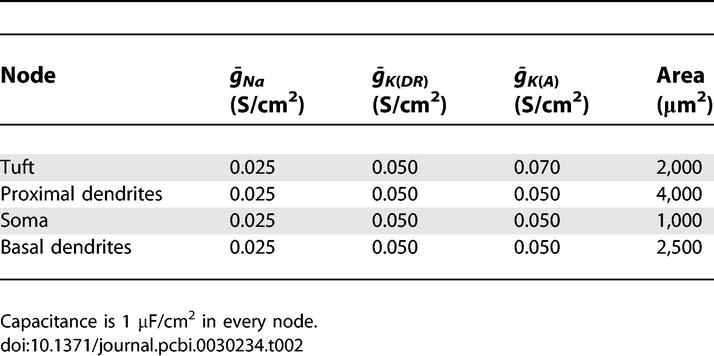
Further Parameters of CA1 Pyramidal Cell Models

The current, *I*, on the right hand side of Equations 1 and 2, has three components:

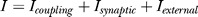



At every time step in the simulations, the voltage of every node is checked and the currents are calculated and added to the derivative.

The four nodes composing each CA1 neuron are connected electrically. The coupling current is calculated from the voltage difference between two nodes and a coupling conductance (Table 3) using Ohm's law:

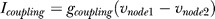



**Table 3:**
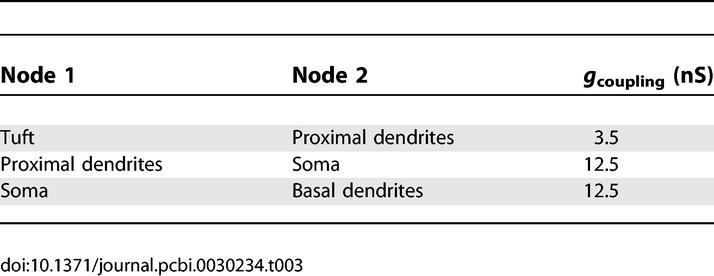
Parameters for Electrical Coupling between CA1 Nodes

Nodes can also be connected with synapses. When the voltage of a node exceeds a threshold of −30 mV, it is said to have generated an event, an action potential, at time *t_event_*. This creates a synaptic current that is added to the derivative of voltage:

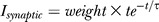
where *t* is measured from the time of the event. Synapses are modeled as alpha functions and have a time constant of 5–20 ms (Table 4). For computational efficiency, events that happen more than 50 ms in the past are not considered. Since the alpha function approaches zero at large *t*, the resulting synaptic current would be negligible. The selection of synaptic weights is discussed in the main text and they are listed in Table 4.

**Table 4:**
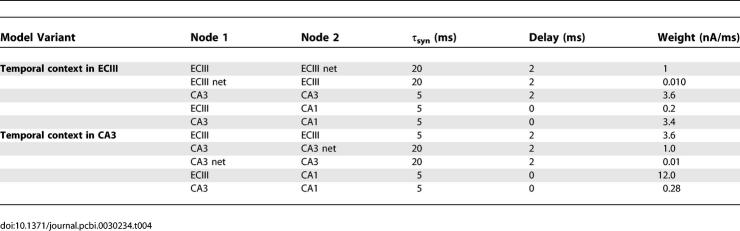
Parameters of Synapse Model

At various points in the simulation, cells receive external current inputs. These inputs are 2-ms current pulses ranging between 100 and 200 pA, also discussed below.

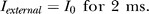



Numerical integration of Equations 1 and 2 is performed using the fourth-order Runge-Kutta algorithm with a time step of 0.001 ms.

All code was written in C and run on a Mac PowerPC with OS 10.4.

## Supporting Information

Video S1 Rat Movie Multimedia clip for Web site.
(3.5 MB MOV)

## Abbreviations

ECIII: layer III entorhinal cortex
PPC: primary place cell
TCC: temporal context cell
